# Genetic risk prediction of the plasma triglyceride response to independent supplementations with eicosapentaenoic and docosahexaenoic acids: the ComparED Study

**DOI:** 10.1186/s12263-020-00669-x

**Published:** 2020-06-15

**Authors:** Bastien Vallée Marcotte, Janie Allaire, Frédéric Guénard, Juan de Toro-Martín, Patrick Couture, Benoît Lamarche, Marie-Claude Vohl

**Affiliations:** 1 Centre Nutrition, Santé et Société-Institut sur la nutrition et les aliments fonctionnels (Institute of Nutrition and Functional Foods (INAF)), Université Laval, 2440 Hochelaga Blvd., Quebec City, Quebec Canada; 2 CHU de Québec Research Center-Endocrinology and Nephrology, Quebec City, Quebec Canada

**Keywords:** Genetic risk score, Plasma triglyceride levels, Omega-3 fatty acids, EPA, DHA, Nutrigenetics

## Abstract

**Background:**

We previously built a genetic risk score (GRS) highly predictive of the plasma triglyceride (TG) response to an omega-3 fatty acid (n-3 FA) supplementation from marine sources. The objective of the present study was to test the potential of this GRS to predict the plasma TG responsiveness to supplementation with either eicosapentaenoic (EPA) or docosahexaenoic (DHA) acids in the Comparing EPA to DHA (ComparED) Study.

**Methods:**

The ComparED Study is a double-blind, controlled, crossover trial, with participants randomized to three supplemented phases of 10 weeks each: (1) 2.7 g/day of DHA, (2) 2.7 g/day of EPA, and (3) 3 g/day of corn oil (control), separated by 9-week washouts. The 31 SNPs used to build the previous GRS were genotyped in 122 participants of the ComparED Study using TaqMan technology. The GRS for each participant was computed by summing the number of rare alleles. Ordinal and binary logistic models, adjusted for age, sex, and body mass index, were used to calculate the ability of the GRS to predict TG responsiveness.

**Results:**

The GRS predicted TG responsiveness to EPA supplementation (*p* = 0.006), and a trend was observed for DHA supplementation (*p* = 0.08). The exclusion of participants with neutral TG responsiveness clarified the association patterns and the predictive capability of the GRS (EPA, *p* = 0.0003, DHA *p* = 0.01).

**Conclusion:**

Results of the present study suggest that the constructed GRS is a good predictor of the plasma TG response to supplementation with either DHA or EPA.

**Trial registration:**

ClinicalTrials.gov, NCT01810003. The study protocol was registered on March 4, 2013.

## Introduction

Supplements of omega-3 fatty acids (n-3 FA) from marine sources, namely eicosapentaenoic and docosahexaenoic acids (EPA and DHA), can be used as an effective, safe, and accessible treatment option for hypertriglyceridemia [1–5]. However, an important inter-individual variability in the plasma triglyceride (TG) response to n-3 FA supplements had been reported, with 29 to 31% of participants increasing TG levels following an n-3 FA supplementation at pharmacological doses [6–8]. This important heterogeneity in the plasma TG response to an n-3 FA supplementation has been shown to be attributable, at least partly, to genetic variations [9]. Considering that TG levels are modulated by a wide variety of factors, and that the physiopathology of hypertriglyceridemia is also quite complex, genetic factors implicated in the regulation of TG levels and the TG response to an n-3 FA supplementation may be various and abundant as well [10–13].

Genetic risk scores (GRS), or polygenic risk scores, have the advantage of pooling the additive effect of many unrelated genetic variations on one trait [14, 15]. Even though they are most commonly used to predict clinical phenotypes [15, 16], we recently demonstrated their applicability to predict the response to nutritional interventions [17–19]. In the Fatty Acid Sensor (FAS) Study, we built a GRS that is highly predictive of the plasma TG response to an n-3 FA supplementation (1.9–2.2 g of EPA and 1.1 g of DHA) in a sample of French Canadians from the province of Quebec (Canada) [18]. This GRS explained 49.7% of the variance of the TG response [18]. However, the contribution of genetic variants to the plasma TG response following a supplementation of either EPA or DHA has never been investigated. Moreover, the TG-lowering effect has been shown to be more important with DHA than with EPA [8, 20], thus emphasizing the importance to better understand the underlying mechanisms, including the effects of genetics.

The independent effects of EPA and DHA were recently investigated in the Comparing EPA to DHA (ComparED) Study, a double-blind, randomized, crossover, controlled trial that aimed to compare the effects of EPA and DHA on inflammatory markers and plasma lipids [21]. The aims of the present study were to further validate the robustness of the GRS previously constructed in the FAS Study in an independent study and to assess its potential to predict the plasma TG response to either EPA or DHA supplementations.

## Materials and methods

### Study design and diets

The study design has been previously described [21]. Briefly, the ComparED Study is a double-blind, randomized, controlled, crossover intervention of three treatment phases: (1) 2.7 g/day of EPA, (2) 2.7 g/day of DHA, and (3) 3 g/day of corn oil as control (0 g of neither EPA nor DHA). Treatments had a median duration of 10 weeks with 9-week washouts between each treatment. Supplements were provided by Douglas Laboratories as re-esterified triglycerides. Throughout the study, participants were asked to maintain a stable body weight, physical activity, alcohol consumption (max. 2 servings/day), and natural health product and vitamin supplement consumption. Alcohol consumption and physical activity were however forbidden 4 days prior to each blood sampling. Participants were also asked to exclude food rich in n-3 FA, such as fatty fish and fish oil supplements, among others, from their diet. Compliance was ensured by the counting of returned unused supplement capsules. Allocation to treatments was concealed to both study coordinators and participants.

### Population

A total of 154 participants were randomized and participated in the study. Participants were recruited at the Institute of Nutrition and Functional Foods (Quebec, Canada) using announcements in newspapers, radio, and electronic newsletters [21]. Subjects had to be between 18 and 70 years old and to have a stable body weight for at least 3 months prior to the randomization. To be eligible for the study, participants had to have abdominal obesity (waist circumference ≥ 80 cm for women and ≥ 94 cm for men) and to present subclinical inflammation defined as plasma CRP levels between 1 and 10 mg/l, exclusively. Women using contraceptive agents were eligible. All participants signed an informed consent form at the beginning of the study approved by the local ethics committees. The study protocol was registered at ClinicalTrials.gov (NCT01810003) on March 4, 2013.

### Anthropometric measurements and blood samples

Anthropometric parameters were measured at screening, before and after each treatment phase following standardized procedures [21]. Blood samples were collected after a 12-h overnight fast at screening, before and after each treatment phase.

### SNP selection and genotyping

The 31 SNPs used in the construction of the GRS in the FAS Study were selected [18]. Details of SNP selection have previously been published [18]. Briefly, SNPs were identified as significantly associated to the TG responsiveness following an n-3 FA supplementation in a previous genome-wide association study (*p* < 1 × 10^−5^) and in a subsequent fine-mapping study [18, 19].

SNPs were genotyped in 123 participants of the ComparED Study using TaqMan technology. The GenElute Gel Extraction Kit (Sigma-Aldrich Co., St. Louis, MO) was first used to extract genomic DNA (gDNA) from blood samples. Validated primers were mixed with 2.5 μl of OpenArray Genotyper Master Mix (Life Technologies, Carlsbad, CA) and 2.5 μl of each gDNA (40 ng/μl) in a 384-well plate. The mix was loaded onto genotyping plates with the QuantStudio™ 12K Flex OpenArray® AccuFillTM System (Life Technologies). Genotyping was performed using the QuantStudio™ 12K Flex Real-Time PCR System (Life Technologies). Results were analyzed in TaqMan Genotyper v1.3 (Life Technologies). SNPs that could not be genotyped were replaced by SNPs in linkage disequilibrium (LD). Seven SNPs taken from the GRS of the FAS Study, namely rs6966968, rs78943417, rs1216346, rs6933462, rs79624996, rs184945470, and rs10009535, were unavailable for genotyping and were respectively replaced by the following SNPs in LD: rs6951762 (LD 73%), rs10224945 (LD 100%), rs28437435 (LD 91%), rs2050017 (LD 100%), rs11025436 (LD 97%), rs1216349 (LD 96%), and rs13137813 (LD 100%). LD between SNPs was assessed using the web-based application LDlink [22].

### Statistical analysis and genetic risk score

Given that the study is based on a crossover design, post-treatment values following EPA and DHA supplementations were compared to the post-control phase in SAS statistical software v9.4. Changes in plasma TG levels in response to DHA and EPA supplementations (ΔTG) were calculated using mean post-intervention TG levels minus mean TG levels after the control phase. The control phase had no effect on mean TG levels, and TG levels were similar at baseline in the three treatments. Each participant therefore had two ΔTG, one for the DHA supplementation and one for the EPA supplementation.

Participants were classified into subgroups of TG responsiveness according to their ΔTG following DHA and EPA supplementations. The intra-individual variation of plasma TG levels was taken into account to classify participants into subgroups of TG responsiveness [8]. The mean intra-individual variation of plasma TG levels was ± 0.25 mmol/l in the ComparED Study based on the standard deviation of four repeated off-treatment TG measurements. For the purpose of the present study, participants among whom the changes in plasma TG levels following EPA or DHA supplementation remained within this ± 0.25 mmol/l-window were defined as non-responders (NR), participants with a reduction in plasma TG levels greater than 0.25 mmol/l were defined as responders (R), and participants showing an increase in plasma TG levels greater than 0.25 mmol/l were defined as adverse responders (AR).

Hardy-Weinberg equilibrium of SNPs was assessed using a chi-squared test in the PLINK software [23]. SNPs that did not respect HWE were excluded from statistical analyses. Rare allele frequencies (MAF) of participants of the FAS Study were compared with MAF of participants of the ComparED Study. Differences in MAF distribution were also calculated between the subgroups of TG responsiveness.

A GRS was computed by the addition of rare alleles of each participant. Rare alleles were defined according to their odds ratio (OR) of association to the TG response. When the OR > 1, the rare allele had a value of + 1, and when the OR < 1, the rare allele had a value of − 1. Major alleles had a value of 0. In SAS v9.4, ordinal and binary logistic models, adjusted for age, sex, and body mass index (BMI) were used to assess the ability of the GRS to classify participants into subgroups of TG response. Significance was set at *p* < 0.05.

## Results

Detailed characteristics of participants were previously reported [8, 21]. From the 154 participants who were randomized in the ComparED Study, 122 completed all treatment phases and had available TG data and genotypes. Characteristics of participants’ pre- and post-supplementations are shown in Table 1. As determined by the inclusion criteria, participants had abdominal obesity and slightly elevated plasma CRP levels (between 1 and 10 mg/l). Before the supplementation, participants had mean plasma TG levels in the normal range (< 1.7 mmol/l). TG levels were significantly reduced by the EPA and DHA supplementations (13.3% and 18.9%, respectively, *p* < 0.0001), but more so with DHA (*p* < 0.05 between treatments) [21].

**Table 1:** Characteristics of participants pre- and post-supplementations (*n* = 122)

	EPA supplementation			DHA supplementation		
	Pre^a^	Post^a^	*p*	Pre^a^	Post^a^	*p*
Age	53.5 ± 14.7	–	–	53.5 ± 14.7	–	–
Waist circumference (cm)	100.5 ± 10.8	100.6 ± 10.5	0.77	100.5 ± 10.8	100.3 ± 11.1	0.57
Body mass index (kg/m^2^)	29.3 ± 4.2	29.3 ± 4.2	0.99	29.3 ± 4.2	29.3 ± 4.3	0.94
Triglycerides (mmol/l)	1.43 ± 0.71	1.24 ± 0.55	< 0.0001	1.43 ± 0.71	1.16 ± 0.51	< 0.0001
C-reactive protein (mg/l)^b^	3.87 ± 4.31	3.54 ± 3.05	0.33	3.87 ± 4.31	3.29 ± 2.85	0.10

^a^Mean value ± standard deviation

^b^One participant with unavailable CRP levels was excluded (*n* = 121)

Genotyped SNPs are listed in Table 2. One SNP, rs28473103, was not in HWE in this sample and was therefore excluded from statistical analyses, leaving a total of 30 SNPs for GRS construction. Four SNPs had a significantly different MAF between the FAS and the ComparED Study. Seven SNPs (rs61569932, rs1990554, rs114348423, rs75007521, rs117114492, rs143662727, and rs76015249) had a MAF < 5% in the ComparED Study.

**Table 2:** Rare allele frequency comparison of genotyped SNPs between the ComparED and the FAS studies

GWAS locus	Genotyped SNP	Rare allele frequency		Chi-squared	*p*
		ComparED Study	FAS Study		
*IQCJ*	rs62270407	0.30	0.28	0.28	0.59
*IQCJ*	rs7639707	0.06	0.043	0.58	0.45
*NXPH1*	rs12702829	0.34	0.43	4.0	**0.046**
*NXPH1*	rs1837523	0.24	0.26	0.25	0.62
*NXPH1*	rs1990554	0.01	0.01	0.03	0.87
*NXPH1*	rs6951762^a^	0.14	0.16	0.23	0.63
*NXPH1*	rs10224945^a^	0.11	0.10	0.35	0.55
*NXPH1*	rs28473103^b^	0.34	0.36	0.24	0.63
*NXPH1*	rs28673635	0.16	0.15	0.10	0.75
*NXPH1*	rs293180	0.11	0.10	0.003	0.96
*NXPH1*	rs61569932	0.004	0.01	0.80	0.37
*NXPH1*	rs6463808	0.14	0.18	1.19	0.27
*PHF17*	rs114348423	0.008	0.02	1.52	0.22
*PHF17*	rs28437435^a^	0.16	0.35	25.65	**4.1 × 10**^−**07**^
*PHF17*	rs75007521	0.02	0.029	0.89	0.34
*MYB*	rs210962	0.20	0.25	1.79	0.18
*MYB*	rs2050017^a^	0.15	0.15	0.02	0.90
*MYB*	rs72560788	0.08	0.09	0.09	0.76
*MYB*	rs72974149	0.08	0.09	0.38	0.54
*NELL1*	rs117114492	0.02	0.02	0.05	0.83
*NELL1*	rs78786240	0.08	0.03	8.07	**0.005**
*NELL1*	rs1850875	0.46	0.42	0.91	0.34
*NELL1*	rs11025436^a^	0.19	0.13	3.07	0.08
*SLIT2*	rs143662727	0.05	0.02	3.02	0.08
*SLIT2*	rs16869663	0.07	0.07	0.08	0.77
*SLIT2*	rs1216349^a^	0.31	0.11	34.25	**4.9 × 10**^**−09**^
*SLIT2*	rs61790364	0.24	0.18	2.76	0.10
*SLIT2*	rs13137813^a^	0.50	0.45	1.55	0.21
*SLIT2*	rs73241936	0.20	0.15	1.98	0.16
*SLIT2*	rs76015249	0.01	0.011	0.03	0.87
*SLIT2*	rs10009109	0.44	0.45	0.02	0.88

^a^Replacement SNP in linkage disequilibrium with a SNP of the genetic risk score constructed in the FAS Study

^b^SNP not in Hardy-Weinberg equilibrium

SNPs showing significant differences in allele frequency between the subgroups of TG responsiveness are the following: R vs NR: rs12702829 (EPA, MAF = 0.39 among R and 0.22 among NR, *p* = 0.009); NR vs AR: rs117114492 (DHA, MAF = 0 among NR and 0.11 among AR, *p* = 0.0002) and rs1990554 (DHA, MAF = 0 among NR and 0.06 among AR, *p* = 0.01); R vs AR: rs12702829 (DHA, MAF = 0.31 among R and 0.56 among AR, *p* = 0.04; EPA, MAF = 0.22 among R and 0.5 among AR, *p* = 0.007) and rs117114492 (DHA, MAF = 0.02 among R and 0.11 among AR, *p* = 0.04).

**Fig. 1:**
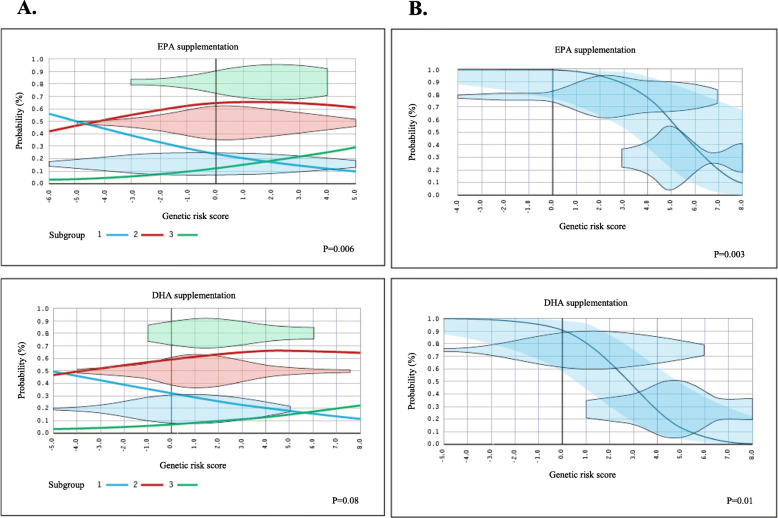
Predicted probability of classification into each subgroup of triglyceride responsiveness following EPA or DHA supplementation. The predicted probability of classification into each of the subgroups of triglyceride responsiveness (R/NR/AR) according to the genetic risk score is represented by curves. Predicted probabilities were assessed by ordinal (**a**) and binary (**b**) logistic regression, adjusted for age, sex, and body mass index. The observed density distribution of participants according to their genetic risk score is illustrated by horizontal violin plots, which are independent of the *y*-axis. **a** All the participants are included in the genetic risk model: (1) responders (R), (2) non-responders (NR), and (3) adverse responders (AR). **b** Only R (upper violin) and AR (lower violin) participants are included in the genetic risk model (NR participants are excluded). The 95% confidence intervals are highlighted in blue

The ability of the genetic risk model to classify participants in the right subgroup of TG responsiveness (NR, R, or AR) was first assessed by ordinal logistic regression, adjusted for age, sex, and BMI. Predicted and observed classifications of participants in the subgroups are presented in Fig. 1. The GRS was associated with TG responsiveness for EPA supplementation (odds ratio (OR) = 1.2, *p* = 0.006), and a trend was observed for DHA supplementation (OR = 1.2, *p* = 0.08). The probability of being classified in the R subgroup increased with lower GRS values, as opposed to the probability of being classified in the AR or NR subgroup (Fig. 1a). Taking NR participants out of the calculation, and using a binary logistic model instead of an ordinal logistic model, resulted in a significant association between the GRS and the subgroup of TG responsiveness for both EPA and DHA supplementations (OR = 2.3, *p* = 0.003; OR = 2.4, *p* = 0.01, respectively) (Fig. 1). In other words, increasing GRS values enhanced the risk of belonging to the AR group.

## Discussion

The primary aim of the present study was to test the applicability of a GRS of the plasma TG response to an n-3 FA supplementation previously built in the FAS Study, in the ComparED Study. In the FAS Study, 208 healthy, non-smokers participants, recruited in the Quebec City metropolitan area, completed a 6-week supplementation of 3 g of n-3 FA. Participants were aged between 18 and 50 years old and had a BMI between 25 and 40 kg/m^2^. Similarly, participants in the ComparED Study were recruited in Quebec City, were aged between 18 and 70 years old, had a pre-supplementation BMI of 29.3 ± 4.2 kg/m^2^, and were healthy. In contrast to the FAS Study, in which participants were supplemented with fish oil containing both DHA and EPA, participants of the ComparED Study were supplemented with either DHA or EPA separately in a crossover study design.

As shown in Fig. 1, the GRS proved to be effective to classify subjects into the right subgroup of TG response (when NR are excluded). However, when all groups of responders were included in the genetic risk model, the association pattern was clearer for the EPA supplementation than for the DHA supplementation. As expected, excluding participants with TG response within the normal variation range resulted in a marked clarification of the association pattern with the GRS for both supplementations. Similar observations were reported in a previous study by our research group, in which a GRS of the plasma TG response to an n-3 FA supplementation at pharmacological doses was computed in a population of Mexicans [17]. In that study, we observed an increasing proportion of TG variance explained by the GRS as participants with the lowest magnitude of TG response were removed from the genetic risk calculation, until the contribution of the GRS reached 29.1% of the TG variance.

Despite demonstrating a good predictive capacity, the results of the present study are not as strong as those reported in the FAS Study. Even though a better prediction is usually expected in the original cohort than in the replication cohort, several factors may have weakened the genetic risk model in the present study, and therefore influenced its predictive capacity. Four main limitations were identified in the present study.

Firstly, seven of the 31 SNPs originally included in the GRS had a MAF < 5% in the ComparED Study. This may also explain why association studies were rather inconclusive with few SNPs showing significantly different allele frequencies between the subgroups of TG responsiveness.

Secondly, the final sample of SNPs included in the genetic risk model was not optimal. Several SNPs included in the original genetic risk model had to be replaced with SNPs in LD with selected SNPs. In the final SNP selection, differences in allele frequency distribution between the FAS and the ComparED populations were observed for four SNPs, two of them being replacement SNPs. Finally, one SNP was removed from the model for not respecting the HWE, as aforementioned.

Thirdly, it is possible that the genetic risk model offers a better prediction when DHA and EPA supplements are taken together rather than separately, perhaps because of a better efficacy of DHA and EPA supplements taken simultaneously in a fish oil extract than supplements of isolated FA. In other words, supplementation of a food extract, as used in the FAS Study (supplements containing DHA and EPA in whole fish oil extract), might trigger stronger gene-diet interactions than supplementation of isolated FA (supplements containing either DHA or EPA), as used in the ComparED Study. It has been previously reported in the literature that the effect of isolated nutrients in supplementation is sometimes different or less effective than the effect of whole food containing the said nutrients [24–27]. More studies investigating the contributive effect of genetic variants on the heterogeneity of the metabolic response to a nutritional intervention should focus on genetic interactions with whole food over isolated nutrients.

Fourthly, the statistical power may have been insufficient. It was previously calculated that a sample size of 150 participants provided 92% statistical power to detect a 10% difference in plasma TG levels between treatments at *p* = 0.01 [8]. The present analyses are based on 122 participants. Moreover, after assignment to subgroups defined on the basis of TG responsiveness, some subgroups had a low number of participants, especially the AR subgroup, which included 9 and 12 AR to DHA and EPA, respectively.

In the present study, the genetic risk model predicted the TG responsiveness to DHA and EPA. The similarity in the genetic risk prediction to EPA and DHA is coherent with observations previously reported in the ComparED Study, in which the magnitudes of TG lowering of DHA and EPA supplementations were similar among responders [8]. However, it appears that the genetic risk prediction to EPA is clearer than to DHA. A possible explanation for this difference may be that, in the FAS Study, the GRS was originally built from an n-3 FA supplementation providing about twice more EPA (1.9–2.2 g) than DHA (1.1 g) daily. Differential responses of epigenetic markers likely to impact gene expression levels have not been observed in previous studies. Vors et al. observed no difference between EPA and DHA supplementations on the expression of 11 inflammatory genes in blood cells in the ComparED Study [28]. Allaire et al. observed no difference between DHA and EPA on the gene expression of HMG-CoA reductase, LDL-R, SREBP1c, and SREBP2 in blood cells [8]. Some differential effects of DHA in comparison with EPA have nonetheless been observed on various cardiometabolic risk markers [8, 21, 29, 30]. These overall observations may suggest that interactions of DHA and EPA with genetic variants possibly impacting gene expression levels are likely to be very similar. A difference in the predictive capacity of the genetic risk model could have been observed between DHA and EPA if they differentially affected gene expression of TG-related genes, but further research is necessary to test this hypothesis. In previous publications by our group, we showed that the potential mechanism of genetic loci in the GRS may pass through modulation of transcript expression and DNA methylation levels [31]. LD between these SNPs and SNPs in nearby genes may explain several associations as well, and *IQCJ* (Table 2) may contribute to TG levels through calmodulin and its effect on calcitonin [18, 32]. However, for most loci, no clear mechanism of action has emerged yet.

## Conclusion

In conclusion, the GRS successfully predicted the plasma TG response to an n-3 FA supplementation of either DHA or EPA, particularly in individuals with most extreme TG responsiveness.

## Data Availability

Datasets used in this study are available from the corresponding author on reasonable request.
